# Progesterone Suppresses Uterine Contraction by Reducing Odontogenic *Porphyromonas gingivalis* Induced Chronic Inflammation in Mice

**DOI:** 10.3390/biom12081029

**Published:** 2022-07-26

**Authors:** Yuko Teraoka, Jun Sugimoto, Haruhisa Konishi, Hiroshi Miyoshi, Hisako Furusho, Mutsumi Miyauchi, Shunichi Kajioka, Iemasa Koh, Yoshiki Kudo

**Affiliations:** 1 Department of Obstetrics and Gynecology, Graduate School of Biomedical and Health Sciences, Hiroshima University, Hiroshima 734-8551, Japan; juns@hiroshima-u.ac.jp (J.S.); iemasako@hiroshima-u.ac.jp (I.K.); yoshkudo@hiroshima-u.ac.jp (Y.K.); 2 Department of Obstetrics and Gynecology, Miyoshi Central Hospital, Miyoshi 728-8502, Japan; haru.konishi@gmail.com; 3 Department of Obstetrics and Gynecology, Hiroshima Prefectural Hospital, Hiroshima 734-0004, Japan; hmiyoshi36@hph.pref.hiroshima.jp; 4 Department of Oral and Maxillofacial Pathobiology, Graduate School of Biomedical and Health Sciences, Hiroshima University, Hiroshima 734-8551, Japan; furusyou-1217@hiroshima-u.ac.jp (H.F.); mmiya@hiroshima-u.ac.jp (M.M.); 5 Department of Pharmaceutical Sciences, School of Pharmacy at Fukuoka, International University of Health Welfare, Fukuoka 812-8582, Japan; kajioka@iuhw.ac.jp

**Keywords:** preterm birth, odontogenic infection, chronic inflammation, progesterone, *Porphyromonas gingivalis*

## Abstract

Preterm birth is one of the most significant obstetric complications. Inflammation reportedly promotes uterine contraction and weakening of the fetal membrane, which induces preterm birth. Previous studies using animal models of lipopolysaccharide-induced acute inflammation have shown that progesterone (P4) promotes uterine quiescence. However, this effect is not fully understood in chronic inflammation. This study aimed to investigate the effects of P4 on uterine contractility and inflammation of the fetal membrane in mice infected with *Porphyromonas gingivalis* (*P.g.*), a major periodontal pathogen as a model of preterm birth caused by chronic inflammation. Mice were injected with 1 mg of P4 from day 15.5 to 17.5. P4 prolonged the mean gestation period of *P.g* mice from 18.3 to 20.4 days, and no reduction in the gestation period was observed. P4 treatment suppressed spontaneous uterine contractility and decreased oxytocin sensitivity. In addition, the expression of inflammatory cytokines in the fetal membrane was significantly reduced. Thus, P4 prevented preterm birth by suppressing enhanced uterine contractility induced by chronic inflammation in this model. This result describes the effects of P4 in a chronic inflammation model, which may lead to a better understanding of the efficacy of P4 in preventing preterm birth in humans.

## 1. Introduction

Preterm birth has a significant impact on neonatal morbidity and mortality; hence, preterm birth prevention is one of the most critical goals of perinatal care. Despite tremendous efforts to treat or prevent preterm birth, the rate of preterm birth has remained unchanged; in fact, it has increased slightly in Japan [1] and worldwide [2]. Preterm births frequently have increased uterine contractions, cervical ripening, and premature membrane rupture [2]. 

Reportedly, the clinical symptoms leading to preterm birth are related to maternal inflammation [3]. Acute infections such as chorioamnionitis and urinary tract infections can cause preterm birth. Chronic inflammation also leads to preterm birth and is known to cause many systemic diseases, such as diabetes, nonalcoholic steatohepatitis, and cardiovascular disease [4,5,6]. Periodontal disease (PD) is a chronic inflammatory condition that persists in the oral cavity. Recent studies have indicated that PD is associated with preterm birth and fetal growth restriction (FGR) [7,8,9,10].

We have previously reported a mouse model of infection with *Porphyromonas gingivalis* (*P.g*.), a common pathogen of odontogenic disease. This *P.g.* mouse model has a gestational age of two days shorter and may be useful for evaluating preterm birth due to chronic inflammation [11]. Reportedly, systemic inflammation is already present at the time of mating in the *P.g.* mice, with a marked increase in serum level of cytokines such as IL-1β, TNFα, and IL-6 [12]. In addition, serum IL-1β and TNFα measured on gestational day 18.0 significantly increased compared to the control mice [11]. These results confirmed that the inflammation established before mating persists after pregnancy and that this model is based on chronic inflammation. In the *P.g.* mice, gene and protein expression of contractile-associated proteins (CAPs) was increased on gestational day 18.0 [11]. In addition, prolonged inflammation before pregnancy (chronic inflammation) is thought to promote activation of the myometrium and induce preterm birth. 

Progestin (P4 and 17-OHP (17-hydroxyprogesterone acetate)) administration is clinically used to prevent preterm birth globally. P4, in particular, is known for its ability to inhibit cervical ripening in humans [13]. Although there have been various reports on the effect of P4 in preventing preterm birth using acute inflammation models, the effect of P4 on chronic inflammation is not well understood. 

This study aimed to investigate the effects of P4 on preterm birth using a mouse model of chronic inflammation. We measured the changes in uterine contractility and CAPs expression in the uterine myometrium of *P.g.* mice. We also evaluated the inflammatory cascade in the fetal membrane, the main site of inflammation in *P.g.* mice.

## 2. Materials and Methods

### 2.1. Animal Experiments

Female C57BL/6J mice, eight weeks old, were purchased from Charles River in Japan (Yokohama, Japan). This study was conducted in conformity with the rules of Hiroshima University’s Committee of Research Facility for Laboratory Animal Science (authorization number: E-582-1). The mice were mated overnight, and pregnancy was confirmed by the presence of a vaginal plug. The day when the plug was present was designated as gestational day 0.

### 2.2. Chronic Inflammation-Induced Preterm Birth Model with P.g. Infection 

Odontogenic infection with *P.g.* was performed as described previously [4]. Briefly, a small swab of phosphate-buffered saline containing 10^7^ cells of *P.g.* was embedded in the right and left first molars of the maxilla and sealed using Caviton (G.C. Corporation, Tokyo, Japan). Six weeks later, periodontal ligament granulomas were observed pathologically, and the serum inflammatory cytokines IL-1β and TNF-α were increased by 2- and 2.5-fold, respectively [12]. Upon confirmation of chronic inflammation, mating was initiated 6 weeks after the *P.g.* infection. The mean gestation periods of *P.g.*-infected (*P.g.* mice) and *P.g.*-uninfected (control mice) were 18.3 ± 0.9 and 20.5 ± 0.5 days, respectively. Serum P4 levels and the corpus luteum structure were observed, and we observed that preterm birth in the *P.g.* mice was not caused by P4 withdrawal but by the fetal membrane inflammation [11]. *P.g*. mice had increased gene expression of oxytocin receptors and connexin 43 on day 18.0 of gestation [11]. Therefore, in this study, we compared spontaneous uterine contractility and sensitivity to oxytocin in the *P.g*. mice and *P.g.* + P4 mice on gestational day 18.0 and examined CAPs expression in the myometrium and inflammatory cytokines in the fetal membrane in each group at the same time. 

### 2.3. Method of P4 Administration

The *P.g*. mice were administered 1 mg of P4 by subcutaneous injection for 3 days from day 15.5 to 17.5 of gestation (*P.g.* + P4 mice). 

### 2.4. Tissue Collection

Under anesthesia, pregnant mice were sacrificed, and tissues were harvested on day 18.0 of gestation. The uterus was removed, and the placenta, fetal membranes, and uterine muscles were carefully collected.

### 2.5. Measurement of Uterine Smooth Muscle Contractility

Experiments on uterine contraction have been described previously [14]. Briefly, strips of uterine smooth muscle (~5 mm wide and ~7 mm long) were suspended vertically between two steel wires in an organ chamber containing 10 mL of modified Krebs solution bubbled with 5% CO_2_ and 95% O_2_ (pH 7.4, 37 °C) at gestation day 18.0. Spontaneous contractions were measured after a stable response was achieved. Oxytocin (PEPTIDE INSTITUTE, Osaka, Japan) was then added to the tissue organ bath at 20 min intervals at increasing concentrations from 1 pM to 300 nM. A force transducer was used to record uterine contractions (UL2; Minebea Co., Ltd., Osaka, Japan). Power was determined by calculating the area under the contraction curve (AUC) for the final 5 min at each dose and dividing by the weight of the uterine strip. Oxytocin sensitivity was evaluated by the percentage change and expressed as follows: ΔAUC (%) = (AUC each − AUC spontaneous) × 100/AUC max − AUC spontaneous. 

### 2.6. RNA Isolation and Quantitative RT-PCR Analysis 

After homogenization, total RNA was isolated from tissue specimens using an RNeasy mini kit (74134; Qiagen, Valencia, CA, USA) according to the manufacturer’s instructions. Spectrophotometry was used to confirm the quantity and purity of RNA. After the genomic DNA was removed from the samples using DNase I (D5307; Sigma-Aldrich, St Louis, MO, USA), reverse transcription was performed using the Omniscript Reverse Transcription Kit (205113; Qiagen, Valencia, CA, USA), as described in the protocol. Total RNA (1 μg) was used for complementary DNA (cDNA) synthesis, which was diluted 10-fold. Real-time PCR was performed using a 7300 Real-Time PCR System (Applied Biosystems, Waltham, MA, USA). One microliter of a 10-fold diluted reverse transcription product was used for real-time RT-PCR with SYBR Green I in a total volume of 25 mL (Applied Biosystems *Power* SYBR Green Master Mix: 4368577; Thermo Fisher Scientific, Waltham, MA, USA). The PCR conditions were as follows: 1 cycle at 95 °C for 10 min, followed by 40 cycles at 95 °C for 15 s and 56 °C for 60 s. Primer sequences were as follows: mouse IL-8 forward:5′-CGC TGC TGC TGC TGG CCA CCA-3′ and reverse:5′-GGT TAT GAC TTC GGT TTG GGT GCA G-3′ [12]. The other gene primers used for real-time PCR have been previously described [11,14]. The expression of each gene was normalized to that of the housekeeping gene, GAPDH. Relative gene expression data were analyzed using the standard curve method. Each sample was analyzed in duplicate (n = 8).

### 2.7. Western Blotting 

Western blot analysis of NF-κB and MAPK was performed in the fetal membrane samples. Tissues were lysed in RIPA buffer (J63306: Thermo Fisher Scientific, Waltham, MA, USA) containing protease inhibitor (165-26021: Fuji film, Osaka, Japan), and 1000 μg of protein extracts were subjected to electrophoresis using 4–12% SDS-PAGE gels (NW04127: Thermo Fisher Scientific, Waltham, MA, USA). Proteins were transferred to membranes using an iBlot2 Dry Blotting System (IB21001: Thermo Fisher Scientific, Waltham, MA, USA). The membranes were incubated overnight with the following primary antibodies: anti-phosphorylated-NF-κB p65 (1:1000; #3033: Cell Signaling Technology (CST), Danvers, MA, USA), anti-NF-κB p65 (1:3000; #8242: CST), anti-phosphorylated-JNK (1:1000; #4668: CST), anti-JNK (1:1000; #9252: CST) anti-phosphorylated-p38 MAPK (1:3000; #4511: CST), anti-p38 MAPK (1:3000; #8690: CST), and anti-cyclophilin B (1:20,000; ab178397: Abcam, Cambridge, UK). Cyclophilin B was used as a loading control. Protein bands were densitometrically analyzed using Image Saver 5 for the Ez-Capture series (ATTO Corporation, Tokyo, Japan). Image J version 1.51 was used to calculate the relative densities of the bands (National Institutes of Health, Bethesda, MD, USA).

### 2.8. Statistical Analysis 

Statcel 3 (Microsoft, Redmond, WA, USA), an add-in software for Microsoft Excel, was used for the statistical analysis of the data. Statistical significance was determined using the *t*-test or Mann–Whitney U test. Statistical significance was set at *p*-value < 0.05.

## 3. Results

### 3.1. Change in the Gestational Period

The average gestation period of the *P.g.* mice was 18.3 days, while that of the *P.g*. + P4 mice was 20.4 days (n = 5), which was the same as that of the control mice, and no reduction in gestation period was observed.

### 3.2. P4 Attenuated Increased Uterine Contractions 

**Figure 1:**
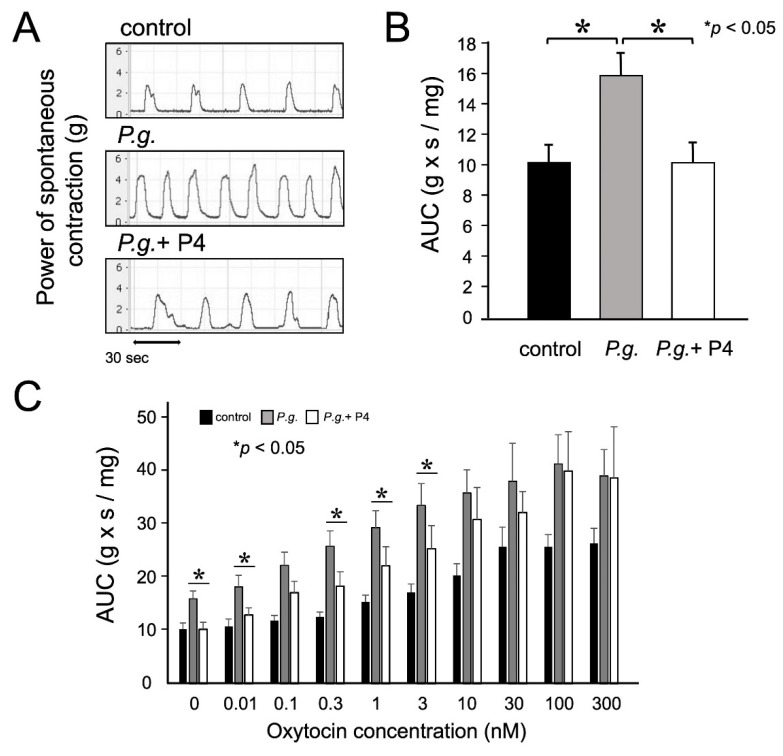
The effects of progesterone (P4) on uterine contraction and oxytocin sensitivity. (**A**) After a stable response was obtained, spontaneous contractions were measured. Uterine contractions were recorded with a force transducer, and the power was determined by calculating the AUC (area under the contraction curve) at the final 5 min. (**B**) The mean AUC of spontaneous contractile activity was decreased by 37% in the *Porphyromonas gingivalis* (*P.g.*) + P4 group (n = 6) compared to the *P.g.* group (n = 8) at day 18.0 of gestation. (**C**) Mean AUC values for uterine contractions induced by various oxytocin concentrations. Oxytocin was added to the tissue organ bath at 20 min intervals in increasing concentrations from 1 pM to 300 nM. Experiments were performed in duplicate using 6–8 independent tissue samples. Statistical analysis was performed using the Mann–Whitney U test. Statistical significance was set at *: *p* < 0.05. Values represent mean  ±  SD (n  = 6–8).

Uterine strips at gestational day 18.0 were used to measure spontaneous contractile activity and sensitivity to oxytocin and were compared between the control, *P.g.* mice, and *P.g.* + P4 mice. The *P.g.* mice tended to have stronger and more frequent uterine contractions than the control group, and the contractions that were enhanced in the *P.g*. mice were attenuated and less frequent in the *P.g.* + P4 mice (Figure 1A). The mean 5-min AUC of the *P.g.* mice was 1.5 times higher than that of the control group but was attenuated by 37% in the *P.g.* + P4 mice (*p* < 0.05, n = 6–8) (Figure 1B). Thus, spontaneous contractile activity, which was enhanced in the *P.g.* group, was attenuated by P4 administration. Next, oxytocin was added to the tissue baths. Oxytocin activated uterine contractions in a concentration-dependent manner in all groups (Figure 1C). Oxytocin concentration-dependent curves were fitted by the minimum square method using ΔAUC values. The half-maximum effective concentration was 3.1 ± 0.7 nM in the control mice, 1.2 ± 0.4 nM in the *P.g*. mice, and 1.9 ± 0.6 nM in the *P.g.* + P4 mice. This result indicated that P4 treatment decreased uterine sensitivity to oxytocin.

### 3.3. P4 Suppressed the Expression of CAPs and L-Type Ca^2+^ Channel in the Myometrium 

The gene expression of contractile-associated proteins and calcium (Ca^2+^) ion channel receptor in the uterine myometrium of the *P.g*. mice was examined on gestational day 18.0. The expression of the oxytocin receptor, connexin 43, PGF2α receptor, and L-type Ca^2+^ channel increased 3.3-, 3.2-, 2.4-, and 2.7-fold, respectively, in the *P.g.* mice compared to in the control mice (*p* < 0.05). P4 administration decreased gene expression of the oxytocin receptor, connexin 43, and L-type Ca^2+^ channels by 70%, 61%, and 70%, respectively (Table 1). We hypothesize that the decreased gene expression of CAPs and L-type Ca^2+^ channel, which are closely related to uterine contraction, led to the suppression of uterine contraction. 

### 3.4. P4 Had an Anti-Inflammatory Effect on the Fetal Membrane and Suppressed Cytokine Gene Expression

To confirm the anti-inflammatory effects of P4, we examined changes in the gene expression of inflammatory cytokines and COX2 in the fetal membrane, which is the main source of inflammation in the *P.g*. mice. The expression of IL-1b, IL-8, TNF-α, and COX-2 in fetal membranes on gestational day 18.0 was investigated. The gene expression of IL-1β, IL-8, TNF-α, and COX2 in the *P.g.* mice increased by 2.3-, 7.2-, 1.9-, and 2.5-fold, respectively. P4 treatment decreased this expression by 72%, 73%, 68%, and 61%, respectively (Table 2).

### 3.5. P4 Suppressed Activated Inflammatory Pathways in the Fetal Membranes

**Figure 2:**
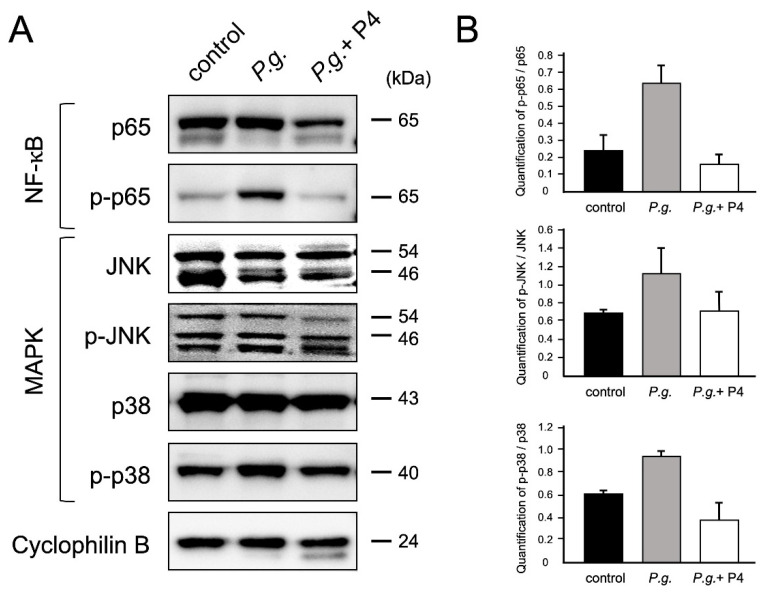
P4 inhibited *P.g*.-induced NF-kB and MAPK phosphorylation (**A**) Western blot analysis was performed to detect the phosphorylation levels of NF-κB and MAPK signaling. Lane 1: control mice; Lane 2: *P.g*. mice; Lane 3: *P.g.* + P4 mice. (**B**) Densitometric data of protein analysis. The phosphorylated/total protein ratio was then quantified. Total and phosphorylated proteins were normalized to cyclophilin B in each group (control, *P.g.*, and *P.g.* + P4). Experiments were performed using three independent tissue samples. Values represent the mean  ±  SD (n  = 3).

We confirmed how P4 administration altered the NF-κB and MAPK pathways activated in the fetal membranes of the *P.g.* mice. Western blot analysis was performed to detect phosphorylated NF-κB p65, pJNK, and p38 MAPK, and activation of these proteins was suppressed by 76%, 38%, and 60%, respectively (Figure 2). Suppression of the NF-κB and MAPK pathways in the fetal membranes was assumed to decrease the gene expression of inflammatory cytokines. 

## 4. Discussion

The global incidence of preterm birth is about 11%, with approximately 15 million children born prematurely yearly [2]. In Japan, the rate is about 6%, with no decrease in view [1]. Prematurity directly affects the life expectancy of preterm infants [2]. Despite advances in medical technology, very early preterm birth is still difficult to save. Inflammation, such as maternal intrauterine infection or chorioamnionitis have been reported to cause preterm birth. Several studies have been performed in animal models treated with bacterial infection, LPS, or inflammatory cytokines [15,16,17,18,19]. However, these acute inflammation models may differ from the clinical situation. Therefore, we performed experiments using *P.g.* mice, a chronic inflammation model.

This study aimed to confirm the effect of P4 on chronic inflammation and prevention of premature birth using *P.g*. mice. In this model, in addition to persistent systemic inflammation prior to conception [11], local inflammatory pathways were activated in the fetal membrane, leading to increased uterine contractions and preterm birth [15]. P4 plays a critical role in maintaining uterine quiescence throughout most pregnancies [20,21]. It is noteworthy that P4 treatment prolonged the gestation period of *P.g*. mice by two days, equivalent to that of the untreated mice. Therefore, we first examined whether P4 reduced uterine contractions in this model, thereby preventing preterm delivery. We also tested the hypothesis that P4 exerts anti-inflammatory effects on the fetal membrane, which is the main source of inflammation.

Spontaneous contractility of the myometrium and its responsiveness to oxytocin were examined using a tissue organ bath system. Spontaneous contractions, which were enhanced in the *P.g*. mice, were attenuated by P4 administration. Generally, increased expression of oxytocin receptors in the myometrium in late pregnancy is known to enhance uterine contractions and contribute to labor onset. The oxytocin concentration-response curve shifted to the right in the *P.g.* + P4 mice, indicating decreased sensitivity to oxytocin. Suppression of uterine contraction by P4 was observed in our chronic inflammation model, as previous reports suggested a genomic effect of contraction inhibition [22,23,24,25]. CAPs are thought to be required for the onset and progression of uterine contractions during parturition [21]. Reportedly, P4 plays an important role in the latter half of pregnancy via anti-inflammatory effects and inhibition of CAPs expression in the myometrium [26,27,28,29]. Oxytocin receptor and connexin 43 expression were significantly reduced by P4 treatment.

Furthermore, progestin is thought to influence uterine contractility through non-genomic and genomic pathways [24,25]. Uterine contraction requires an increase in the intracellular Ca^2+^ concentration, and the L-type Ca^2+^ channel has the greatest effect on Ca^2+^ influx. Inhibition of the L-type Ca^2+^ channel current by P4 has been observed in several previous reports [30,31]. Maternal inflammatory conditions induce increased uterine contraction amplitude, time to peak, and oxytocin sensitivity by increasing endogenous prostaglandin release and Ca2+ influx in L-type calcium channels [32,33,34]. In this study, we did not evaluate the current; however, we observed that Ca^2+^ channel expression was reduced by P4, suggesting that P4 may improve uterine quiescence, including the electrical excitability of myometrial cells. These current results support that P4 suppresses oxytocin sensitivity and CAPs expression in *P.g*. mice, which suppresses uterine contractility, and may be one of the reasons for the delay in labor onset, leading to a prolonged gestation period in the *P.g.* + P4 mice. We then examined inflammation in the fetal membrane that triggered a contraction in the *P.g*. mice. The gene expression of inflammatory cytokines and COX2, the enzyme for PGE2 production, was significantly upregulated in the fetal membrane but was markedly suppressed by P4 administration. 

In addition, activation of NF-kB and MAPK pathway proteins in the fetal membrane was also reduced by P4 administration, suggesting that cytokines via these inflammatory pathways were decreased. The anti-inflammatory effects of P4 have been described as genomic via nuclear receptors and non-genomic via receptors located in the plasma membrane and cytoplasm. Various studies using human amniotic tissue and cells (chorionic villus cells, amnion cells, etc.) have also reported the anti-inflammatory effects of P4 [35,36]. These findings are consistent with our results. The mechanism by which P4 suppresses inflammation is mediated by receptors such as the classical progesterone receptor, membrane receptor, and glucocorticoid receptor [35,36]. Since we only examined the fetal membrane tissue in this study, it would be necessary to investigate the anti-inflammatory effects of P4 at the cellular level in future studies. 

Our study results suggest that P4 may contribute to the prevention of preterm birth by suppressing uterine contractions in a chronic inflammatory model by reducing the inflammation of the fetal membrane. 

In conclusion, this study established the potential effectiveness of P4 in preventing preterm birth induced by chronic inflammation. P4 acted by suppressing the expression of CAPs by inhibiting inflammation of the fetal membrane, leading to the suppression of uterine contractions. However, it is possible that P4 directly affects the expression of CAPs. The effect of P4 on preterm birth was investigated at the molecular level in this study. Further studies on human fetal membranes would help determine the potential of P4 for the clinical treatment of preterm birth in humans.

## Figures and Tables

**Table 1:** Gene expression of contractile associated proteins and calcium ion channel in the myometrium. The gene expression levels of CAPs (oxytocin receptor, connexin 43, and PGF2α) and L-type calcium (Ca^2+^) channel in the myometrium were determined using real-time RT-PCR in each group (control, *Porphyromonas gingivalis* (*P.g.*) and *P.g.* + progesterone (P4)). Experiments were performed in duplicate using eight tissue samples. Statistical analysis was performed using the Mann–Whitney U test. Statistical significance was set at *: *p* < 0.05, compared *P.g.* with the control or: ^†^: *p* < 0.05, *P.g.* + P4 with *P.g.*, respectively. Values represent the mean  ±  SD (n  = 8).

	Control	*P.g.*	*P.g.* + P4
Oxytocin receptor	0.33 ± 0.30	1.10 ± 0.56 *	0.34 ± 0.14 ^†^
Connexin 43	0.34 ± 0.19	1.11 ± 0.50 *	0.44 ± 0.18 ^†^
PGF2α	0.25 ± 0.09	0.61 ± 0.11 *	0.45 ± 0.15
L type Ca^2+^ channel	1.39 ± 0.32	3.79 ± 1.77 *	1.16 ± 0.37 ^†^

* *p* < 0.05 (comparison of *P.g.* with control), ^†^ *p* < 0.05 (comparison of *P.g.* + P4 with *P.g.*).

**Table 2:** Gene expression of cytokines and COX-2 in the fetal membrane. The transcript levels of inflammatory cytokines (IL1b, IL-8, and TNF-α) and COX2 in the fetal membrane were determined by real-time RT-PCR in each group (control, *P.g.*, and *P.g.* + P4). Experiments were performed in duplicate using eight independent tissue samples. Statistical analysis was performed using the Mann–Whitney U test. Statistical significance was set at *: *p* < 0.05, †: *p* < 0.05 compared *P.g.* with the control or *P.g.* + P4 with *P.g.*, respectively. Values represent the mean  ±  SD (n  = 8).

	Control	*P.g.*	*P.g.* + P4
IL-1β	0.55 ± 0.23	1.29 ± 0.47 *	0.37 ± 0.31 ^†^
IL-8	0.51 ± 0.60	3.71 ± 1.46 *	1.02 ± 0.61 ^†^
TNF-α	0.44 ± 0.16	0.85 ± 0.25 *	0.28 ± 0.11 ^†^
COX-2	0.37 ± 0.14	0.93 ± 0.25 *	0.37 ± 0.15 ^†^

* *p* < 0.05 (comparison of *P.g.* with control), ^†^ *p* < 0.05 (comparison of *P.g.* + P4 with *P.g.*).

## Data Availability

The data that support the findings of this study are available from the corresponding author upon reasonable request.
